# A method for detailed determination of hospital surge capacity: a prerequisite for optimal preparedness for mass-casualty incidents

**DOI:** 10.1007/s00068-022-02081-z

**Published:** 2022-09-26

**Authors:** Kristina Lennquist Montán, Per Örtenwall, Magnus Blimark, Carl Montán, Sten Lennquist

**Affiliations:** 1 Department of Global Public Health, Karolinska Institute, Solna, Sweden; 2 University of Gothenburg, Göteborg, Sweden; 3 Centre for Defence Medicine, Swedish Armed Forces, Göteborg, Sweden; 4 Department of Vascular Surgery, Karolinska Institutet, Stockholm, Sweden; 5 University of Linköping, Linköping, Sweden

**Keywords:** Major incident, Mass casualty incident, Disaster, Surge capacity, Hospital capacity, Simulation, Training, Hospital preparedness, Hospital contingency

## Abstract

**Background:**

Defined goals for hospitals’ ability to handle mass-casualty incidents (MCI) are a prerequisite for optimal planning as well as training, and also as base for quality assurance and improvement. This requires methods to test individual hospitals in sufficient detail to numerically determine surge capacity for different components of the hospitals. Few such methods have so far been available. The aim of the present study was with the use of a simulation model well proven and validated for training to determine capacity-limiting factors in a number of hospitals, identify how these factors were related to each other and also possible measures for improvement of capacity.

**Materials and methods:**

As simulation tool was used the MACSIM® system, since many years used for training in the international MRMI courses and also successfully used in a pilot study of surge capacity in a major hospital. This study included 6 tests in three different hospitals, in some before and after re-organisation, and in some both during office- and non-office hours.

**Results:**

The primary capacity-limiting factor in all hospitals was the capacity to handle severely injured patients (major trauma) in the emergency department. The load of such patients followed in all the tests a characteristic pattern with “peaks” corresponding to ambulances return after re-loading. Already the first peak exceeded the hospitals capacity for major trauma, and the following peaks caused waiting times for such patients leading to preventable mortality according to the patient—data provided by the system. This emphasises the need of an immediate and efficient coordination of the distribution of casualties between hospitals. The load on surgery came in all tests later, permitting either clearing of occupied theatres (office hours) or mobilising staff (non-office hours) sufficient for all casualties requiring immediate surgery. The final capacity-limiting factors in all tests was the access to intensive care, which also limited the capacity for surgery. On a scale 1–10, participating staff evaluated the accuracy of the methodology for test of surge capacity to MD 8 (IQR 2), for improvement of disaster plans to MD 9 (IQR 2) and for simultaneous training to MD 9 (IQR 3).

**Conclusions:**

With a simulation system including patient data with a sufficient degree of detail, it was possible to identify and also numerically determine the critical capacity-limiting factors in the different phases of the hospital response to MCI, to serve as a base for planning, training, quality control and also necessary improvement to rise surge capacity of the individual hospital.

## Introduction

The risk for mass-casualty incidents (MCI), situations where the immediate need of resources exceed available capacity to such extent that life and health is in danger, is continuously increasing, parallel to the present development in the world. This puts high demands on the health care system, requiring accurate planning and accurate training [1–3].

A prerequisite for designing both an optimal preparedness and an optimal training is to define the goals for the health care—what to be able to do, and how many casualties to be able to handle with acceptable quality of care. This requires methods to determine the capacity for different units, and also to secure that the defined goals are reached and maintained. This is also a prerequisite for improvement—“what you cannot measure, you cannot improve” [4].

The capacity of a health care unit is commonly defined as *surge capacity* as the “ability to obtain adequate staff, supplies and equipment, structures and systems to provide sufficient care to meet immediate needs caused by an influx of patients following a large-scale incident or disaster” [5–9]. Surge capacity is dependent on several factors and has been described to be based on four domains named as “staff, supplies, space and systems”, all with sub-components [10]. This means that it cannot be calculated only based on resources, but is dependent also on factors like organisation (disaster preparedness) and staff competence (education and training).

Many attempts have been done to calculate surge capacity using mathematical models [11], scoring systems [12] or indexes based on broad surveys [4, 13]. However, none of these methods gives the precise capacity of the individual hospital, since hospitals show a wide variation with regard to size, specialisation, economy, staffing, geographic localization and potential scenarios. This requires methods to test the individual hospital, with sufficient accuracy to numerically determine the capacity-limiting factors. This requires practical tests using methods where the critical capacity limits for the individual hospital, as an effect of all these factors, can be numerically determined for all the different components of the chain of management at different time levels in the chain of response. Very few methods to do this for reasonable costs have so far been described in the literature.

The aims of this study were to:

- test hospital capacity for mass-casualty management using an advanced simulation technique validated for training of mass-casualty response
- identify capacity-limiting factors for different units and components in hospitals
- assess how these factors were related to each other
- describe possible measures to increase hospital capacity.

## Methods

### Simulation system

The system used in this study was MACSIM^®^ (MAss Casualty SIMulation system), used for training in the international MRMI courses (Medical Response to Major Incidents and Disasters) [14, 15]. The system was initially developed for scientific evaluation and comparison of methodologies in MCI response [16] and had at the time for this study been used in more than 10 years with more than 5 000 people trained. It had been scientifically validated for fulfilling its objectives [17]. The system had also been used successfully in a pilot test to determine surge capacity in a major hospital [18].

**Fig. 1:**
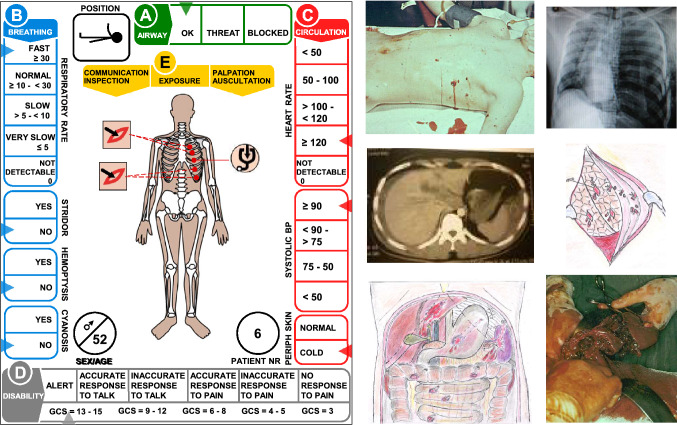
The casualty cards used in this study (for description, see text) were based on real patients from real scenarios. The cards were connected to data files with live pictures, X-ray—and surgical findings as base for decisions with regard to treatment. For each patient, the instructors had access data regarding treatments that had to be done within a certain time to avoid mortality. This made it possible to determine the outcome as a result of the response and of the methodology used

The core component of the system was an extensive number of casualty cards based on real patients and real scenarios (Fig. 1). Along the margins of the card were indicated the patients´ condition with physiological parameters in accordance with ATLS^®^ [19]**,** changing with time after the incident and treatments done/not done. In the central part of the cards, the different injuries were illustrated with a simple system of symbols.

To the cards were connected data files illustrating pictures of injuries, findings at X-ray and surgery, in case it was indicated (Fig. 1), providing a base for decisions with regard to priority and treatment.

**Fig. 2:**
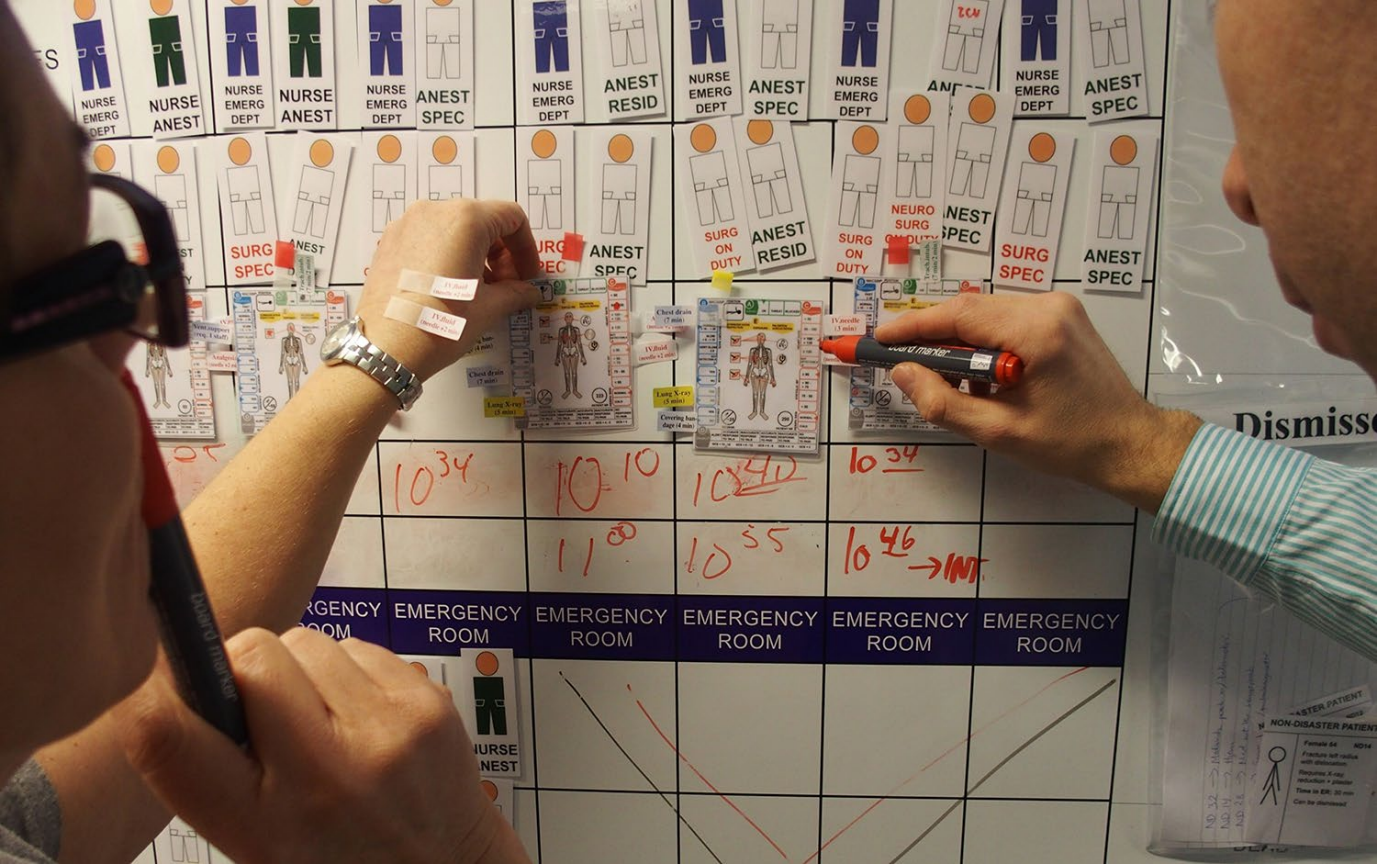
All treatments and major diagnostic procedures were indicated by tags on the cards, also giving the time requested. The tests were run in real time and every treatment had to consume the same time and the same staff in reality. This picture illustrates the activity in the ED

All treatments and major investigations were indicated with movable tags on the cards (Fig. 2), making it possible to determine consumption of all kinds of material. All measures done consumed the same facilities, material, staff and also time required as in reality, and the tests were run in real time.

For each casualty, the facilitators of the test had access to:

- complete injury description (final diagnosis)
- treatments that had to be done for each injury within a certain time to avoid mortality and severe complications
- trauma scores (ISS, NISS, RTS) for each patient
- outcome if treatment had been optimal, to identify preventable mortality.

This altogether made it possible to get a measurable result of the response in preventable mortality and complications.

### Test procedure

Facilities for all involved functions in the tested hospitals were illustrated on a number of pre-fabricated boards of magnetic material, located in a space outside ordinary activities. Participating staff was scheduled for the test, which in this way could be performed without interfering with routine activities.

All ordinary on-going activities had been registered at a time and day corresponding to the day of the test and were illustrated with specific non-disaster patient cards on the boards. All available staff was illustrated by staff symbols, available categories and numbers registered in the same way as above. The tests were run with real consumption of time, facilities, material and staff.

A real scenario was built up on a geographically defined place in the region of the tested hospital. The total number of injured and dead was dead adapted to the local population to achieve a realistic proportion between casualty load and local transport- and health care resources, but the proportions with regard to type and severity of injuries in the scenario were maintained. The scenarios were intentionally exceeding estimated capacity to identify critical limiting factors for all components in the chain of management. The casualty cards were delivered to the hospitals in accordance with available transport resources in the area and with real transport times.

**Fig. 3:**
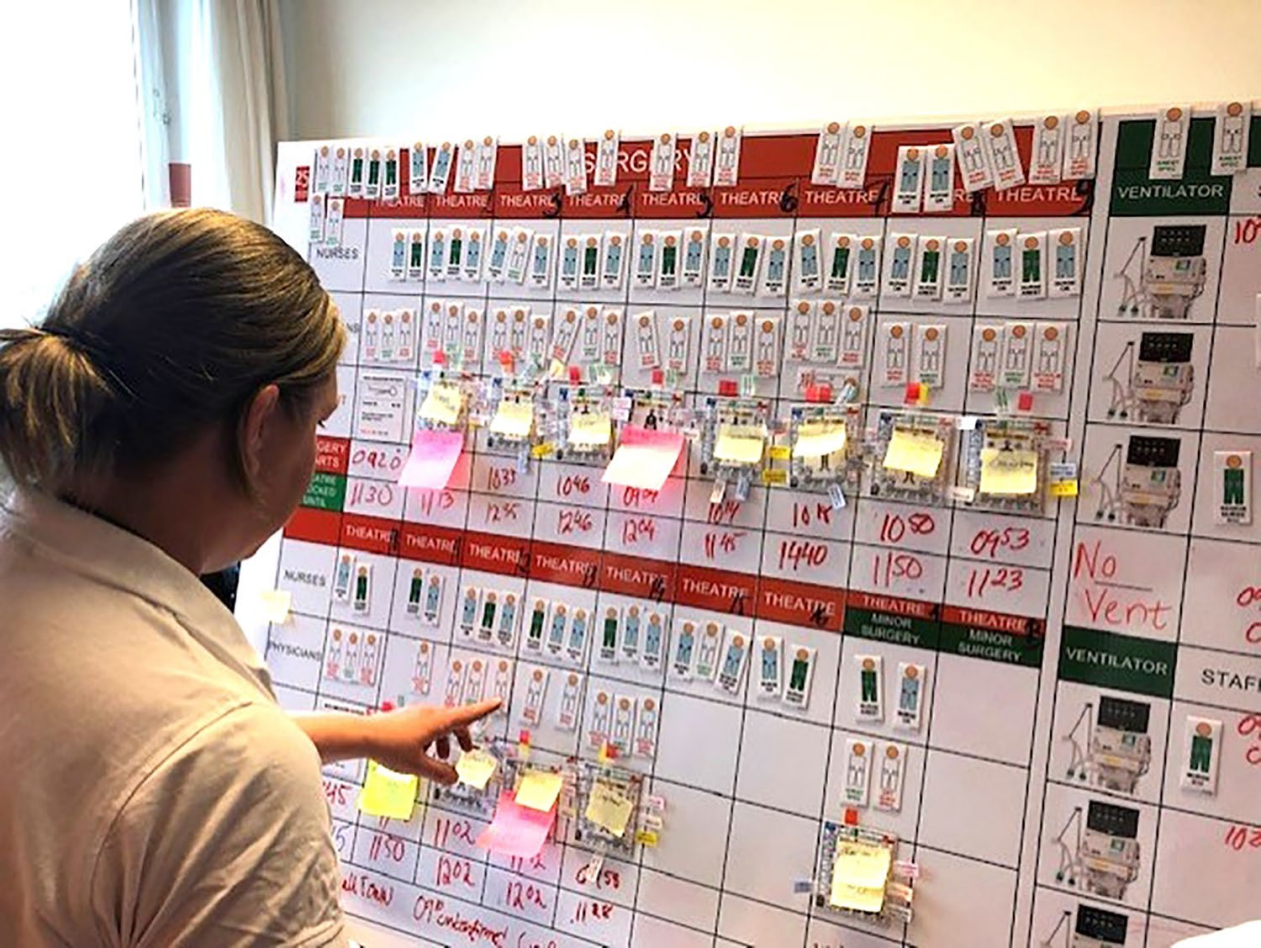
The activity in the operating theatres (OR). All available staff were indicated by staff symbols according to an inventory performed on a corresponding day and time. No action was permitted without accurate staff. The tests were done with real time and no patient could be moved until the time needed for the decided procedures had passed

The casualty cards were registered with the MCI registration system and processed through the hospital by the acting staff in accordance with the disaster plan, including the hospital command group (Fig. 3). The principles for MCI management, including triage and primary management of victims, were in accordance with the principles applied in the international MRMI courses [3] [15].

Specially trained supervisors on each station secured that:

- no treatment was done without consumption of real time and resources of all kinds
- real times were used for change of patients on different positions, and for transport between units
- mortality and complications according to the data in the system due to insufficient resources were continuously registered.

Performed X-rays resulted in preliminary answers and access to pictures. Performed surgery was based on access to pictures from surgical findings and registered in a way that consumption of material (disposable and non-disposable) could be determined.

### Determination of capacity

The number of simulated casualties possible to handle without preventable mortality or complications was defined as the capacity for the different units of the hospital to handle an MCI. Casualties who not could be managed without mortality or complications in lack of resources, or because too long waiting times, were analysed to identify capacity-limiting factors in different positions and in different phases of the response, and also analyse possible measures to reduce or eliminate these factors.

## Statistics

Distribution of the answers in the evaluation form was processed in SPSS Version 22 (IBM, New York, NY) and presented as medians (MD), interquartile ranges (IQR) and minimum–maximum (min–max).

### Ethical considerations

No data from patients or identifiable individuals were included in this study. The fictive “ordinary” patients in the hospital constituted a similar casualty load but were totally changed with regard to age, gender or specific diagnosis. Detailed capacity data, of individual hospitals and units, were not reported due to issues of secrecy and security. All staff participated voluntarily. A request for ethics approval from the Swedish Ethical Review Authority was deemed unnecessary.

## Results

### Performed tests

This study includes totally six tests with the described methodology in three Swedish hospitals (Table 1). Hospitals A and B were University hospitals with all specialities including trauma centres and hospital C was a regional hospital.

**Table 1:** The tested hospitals and test methods

Hospital	Beds, total	Test nr	Year	Time of day	Comments
A	1086	A1* A2	2013 2019	Office hours Office hours	Original hospital New hospital
B	1600	B1 B2 B3	2017 2019 2018	Office hours Non-office hours Office hours	CIMIC**
C	350	C1	2019	Office hours	CIMIC**

*This test was partly described in a previously published pilot study [18]

**Civilian and military co-operation using live figurants transported to the hospital by the military. The figurants were treated in ED and changed to casualty cards with the same injuries for the continued processing in the hospital

Two of the tests, B2 and C1 (Table 1), were initiated by the Swedish Armed Forces Centre for Defence Medicine and the tested hospitals as civilian–military co-operation projects. These exercises started at army bases with live figurants who were moulaged according to the mass-casualty cards, which also were attached to each figurant. The figurants acted accordingly to the casualty cards through the chain of evacuation, transport, ED and X-ray. For the continued processing in the hospital, the figurants were replaced with a smaller version of each casualty card for the continued capacity test of OR, ICU, wards, etc.

Numerical data on capacity cannot be published, since they are classified due to Swedish security regulations.

However, capacity-limiting factors for different components in different phases of the response, and how these factors were related to each other, are reported in chronological order, as a base for identification of possible measures to increase the hospitals surge capacity for MCI.

### Emergency departments

The first factor to limit the hospitals capacity to receive casualties was the number of units required for primary management of major trauma. “Unit” stands for a combination of (a) a room with sufficient space, (b) all necessary equipment, and (c) the minimal number of qualified staff required for this. The number of such units that each hospital could mobilise on alert varied depending on (a) how many regular trauma rooms the hospitals had for routine use and (b) how many such additional units the hospitals had prepared for MCI. Rooms not pre-equipped for this, and staff not fully qualified and trained for major trauma, were not approved.

**Fig. 4:**
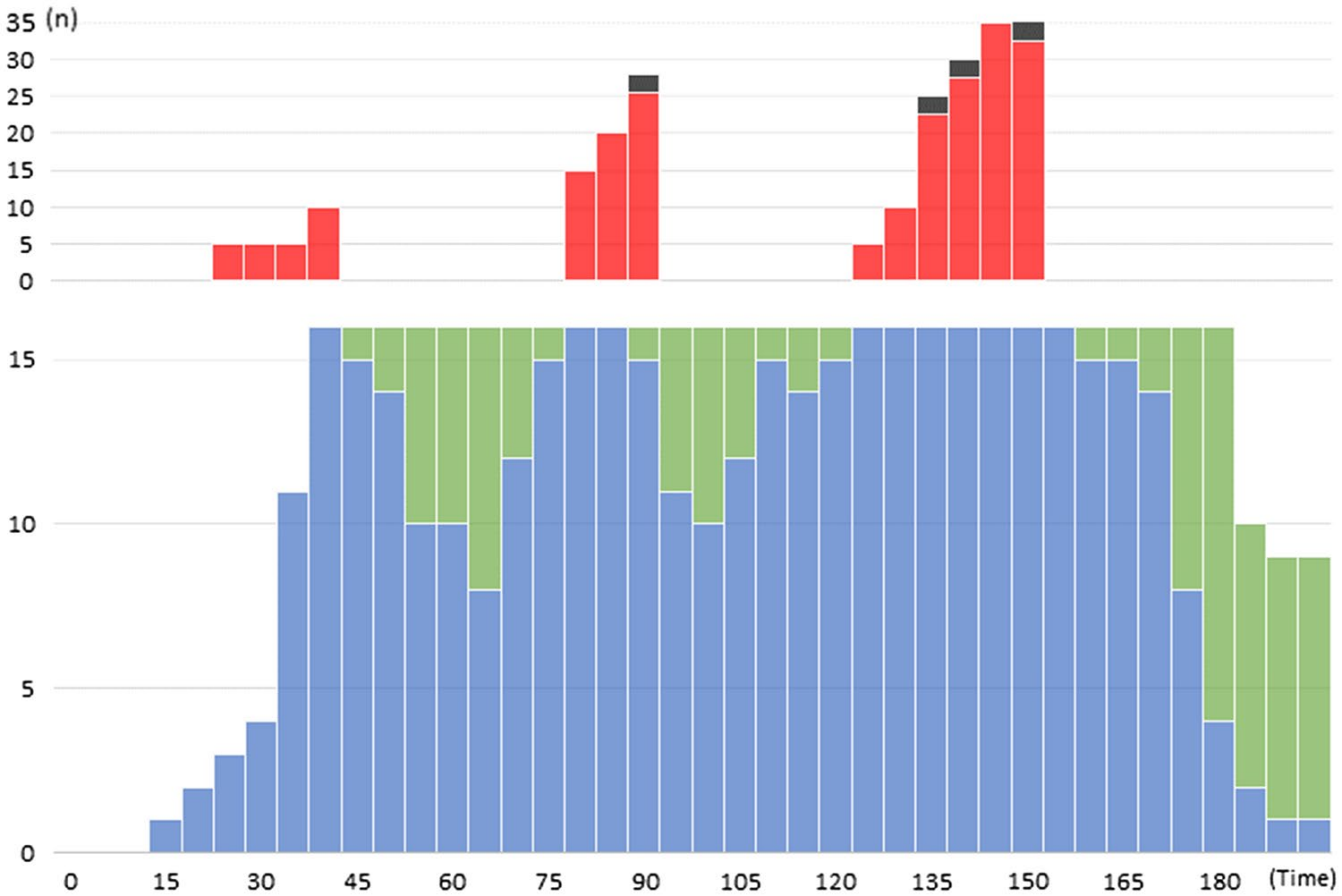
The activation and use of the teams for management of severely injured casualties in the Emergency Department (ED) in one of the tested hospitals (see further the text). The periods of very high casualty load, causing waiting times leading to calculated mortality, correspond to the “waves” of ambulances between returning and re-loading. To avoid preventable mortality, the inflow has to be temporarily stopped and casualties referred elsewhere. This puts high demands on coordination of casualty distribution. Blue: Trauma-teams (modified for MCI) in action, Green: Such trauma teams at disposal, Red: Severely injured patients having to wait for access to teams, Black: Preventable deaths caused by waiting

Figure 4 shows the casualty load in the emergency department (ED) in hospital A before moving into a new hospital building, why these figures are no more relevant for the hospital. The plan for the region at the time of this test was that all severely injured should be sent to this hospital as being the regional trauma centre, and the other hospitals should send staff with training in trauma to this hospital. In this way, it was possible after alert to have 16 parallel teams working with major trauma.

The figure illustrates how the trauma teams were successively activated by incoming patients starting from 15 min after the incident. A natural delay in mobilising teams caused some waiting times in the first “ambulance wave”, culminating 40 min after the incident when capacity was exceeded in spite of full mobilisation of teams. After the first wave, the major trauma rooms could be cleared before the second wave of returning ambulances, this time resulting in an overload of severely injured, with waiting times causing preventable mortality according to the patient data provided in the system. At this time, the inflow was temporarily stopped, but opened again half an hour later when these rooms were cleared. A third wave, now with increasing access to transport resources, overloaded the ED and again caused preventable mortality, i.e., patients which maybe could have been saved with another distribution of patients between hospitals. One learning from this exercise was that sending all severely injured to one hospital not was relevant, even if staff were relocated, and even if the number of rooms prepared for major trauma was high.

A number of less severely injured came to the hospital despite that these patients according to the plan should be sent elsewhere, because of “spontaneous evacuation” from the scene, which is practically un-avoidable in an MCI. However, these patients did not affect the capacity limit, since they did not require specific competence, facilities or equipment. They could also, after re-assessment and re-triage at arrival, wait for treatment without risk for mortality or complications (Fig. 7).

A second test in hospital A was performed after moving to a new hospital building. This was combined with a revision of the hospitals role in the region to be more focused on high degree of specialisation and less general emergency care, resulting in a smaller ED. The test illustrated that this significantly reduced the number of severely injured possible to receive simultaneously in the immediate response. Consequently, it also reduced to possibility to fully utilise the extensive OR and ICU facilities of the hospital, unless it was done by secondary transport after primary management in other hospitals.

**Fig. 5:**
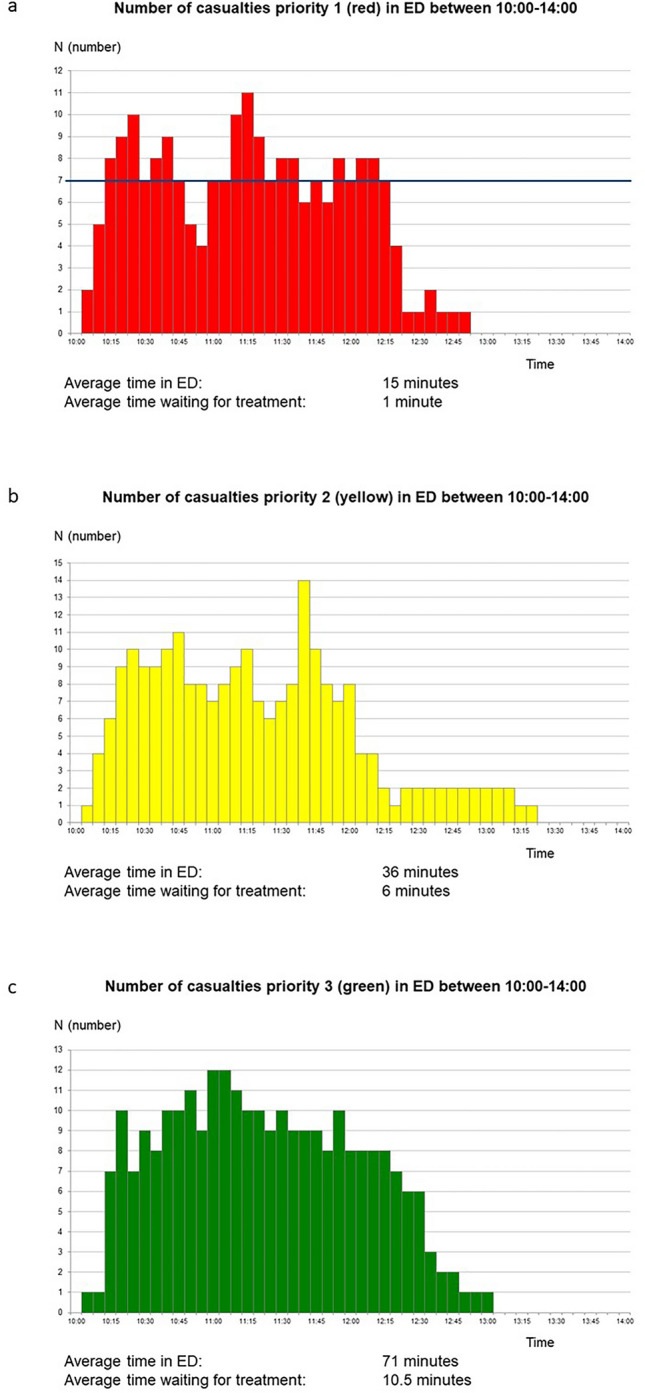
**a–c** The casualty load in hospital B (test B2) for patients triaged as **a** red, **b** yellow and **c** green. *n* = number of patients per time unit. Incident occurred at 10.00. **a** Those triaged as red exceeded the hospitals capacity for major trauma at several occasions, indicated by the black horizonal line, representing the maximal available number of units for major trauma (unit = room with equipment + qualified and sufficient staff). This caused waiting timed leading to preventable mortality according to the patient data provided by the test system. **b** Those triaged as yellow arrived in a similar pattern and number, but because of accurate triage, the waiting times this caused did not lead to any mortality. **c** Those triaged as green arrived in a more continuous flow, all not dependent on ambulances. These patients could be taken care of in other facilities and by other staff, did not interfere with treatment of the more severely injured, and caused no overloading

The other tested hospitals had, different to hospital A, the task to receive patients of all triage categories and not only (as in hospital A) those with red priority. Figure 5a–c illustrates the casualty load on ED in hospital B, showing the same characteristic pattern for patients *triaged as red* (Fig. 5a) as in hospital A with “peaks” related to the return of ambulances after re-loading on scene. During these peaks, the number of teams for major trauma was insufficient, resulting in wating times with risks for preventable mortality and complications according to the patient data provided by the system. To avoid this, these peaks should—as far as possible—be eliminated by referring a number of these patients to other hospitals, requiring an efficient regional coordination.

Those *triaged as yellow* (Fig. 5b) showed peaks similar to the red patients, also leading to waiting times. However, because of relevant triage, this was in no case estimated to lead to mortality or complications.

Those *triaged as green* (Fig. 5c) followed another pattern, more evenly distributed in time, due to the fact that also other transport facilities were used for this category. As mentioned above, most of these patients could be managed in other areas and with other staff and interfered very little with the management of the severely injured.

The casualty load in the ED in hospital C followed the same pattern as in hospital B for all triage categories.

In the first test in hospital B, a need was expressed to determine capacity of the hospital in collaboration with the military, using live figurants labelled with the same casualty cards in larger size, and is described elsewhere [20]. After passing the ED, only the casualty cards were used according to the same methodology as in the other tests. This test was much more resource-consuming, but provided more information with regard to facilities and the logistics in the ED.

### Surgery

**Fig. 6:**
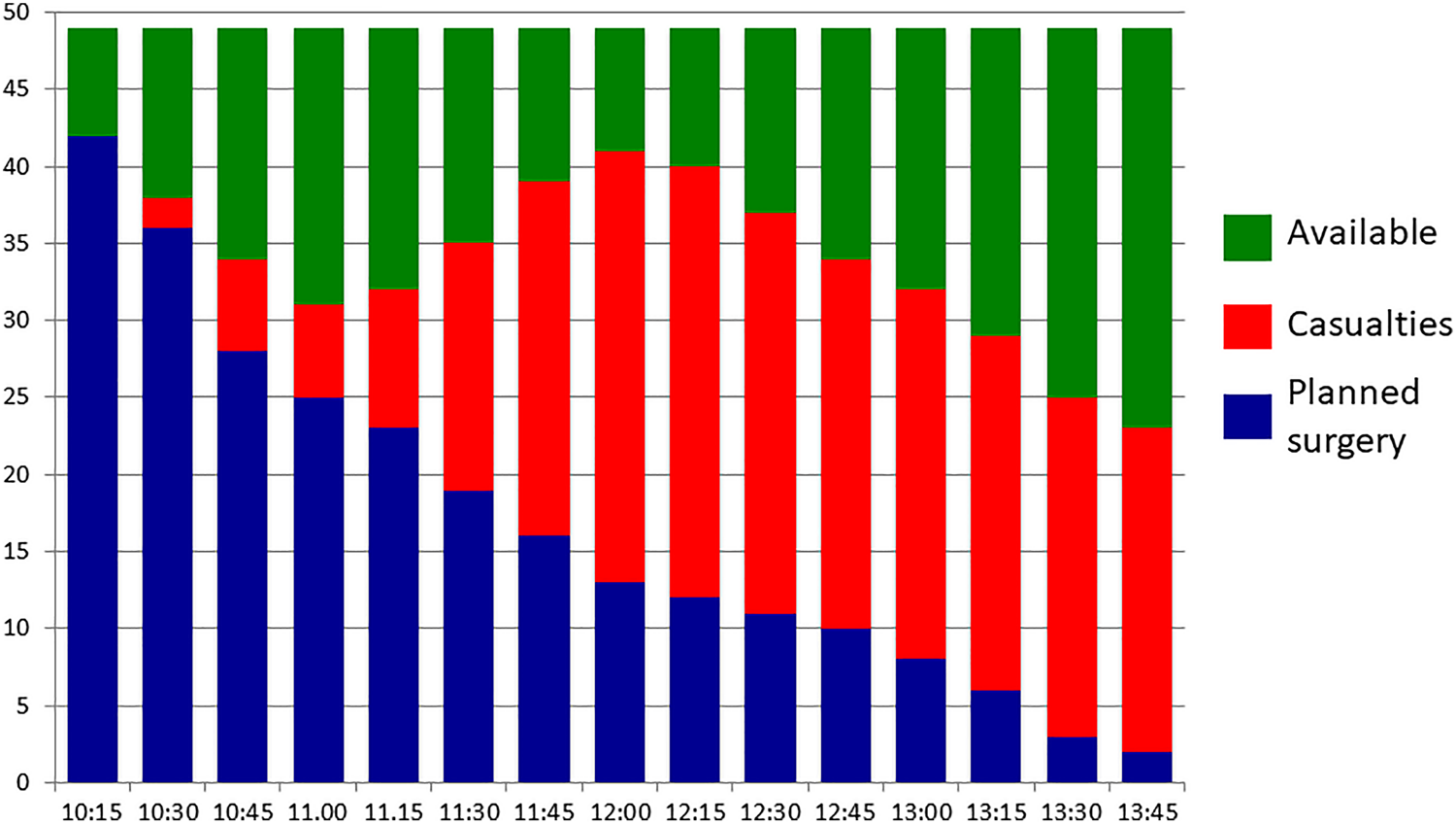
The casualty load on surgery also followed the same pattern in all the tested hospitals, here represented by the first test in hospital A. All hospitals in this test were well equipped with surgical theatres. The figure shows a test during office hours with most theatres occupied by on-going, planned surgery. By immediately on alert stopping all surgery that could wait, theatres needed for MCI patients could be made accessible without delay, and OR capacity was never exceeded. The same was valid in non-office hours, because it was equally fast to get theatres accessible by calling in staff off duty. From Lennquist Montán et al., Eur J Trauma Emerg Surg 2017; 43:525–539, with permission

Figure 6 illustrates the burden on the surgery (OR) in test A1 as described in Fig. 4. The simulated incident here occurred during office hours with full normal activity in all theatres. Still, already when the first casualty arrived in OR 30 min after the alert, there were theatres available, since all planned activity immediately had been stopped at the alert. The figure illustrates how the load of surgery continuously came in a later face that the load on ED.

In spite of the fact that the Madrid 2004 scenario [21] was used for this test with a high load on surgery, the OR capacity of this hospital was never fully utilised, but the limit for surgery was set by the ICU capacity.

Neither in hospital B, the load on surgery exceeded available capacity, since this hospital, as hospital A, had a very good surgical capacity due to many surgical specialties. In the smaller hospital C, every theatre was used on several occasions during the test period, and even if waiting times for immediate surgery could be avoided, there were no margins. If major surgery not had to be stopped because of lack of intensive care, surgery had probably been a capacity-limiting factor in this hospital.

### Intensive care

The ICU capacity was shown to be the definite limiting factor in all these tests, despite sending patients to wards whose treatment could continue there with maintained quality of care, plus transferring patients to other hospitals being assessed to tolerate transport without risk, when staff and transport facilities were available for this. At the time for these tests, the access to reserve-ventilators was very small, and OR ventilators and staff could not be used because of high load of on-going surgery.

### Wards

In none of these tests, the available bed capacity seemed to be a limiting factor, and no “ordinary” patients who could not be safely dismissed from the hospital (as on-going non-urgent investigations, planned elective surgery) needed to be dismissed.

### Serving functions

Laboratory capacity was not exceeded in any of the tests due to restricted use of laboratory capacity. Too liberal use of computer tomography created congestion and non-acceptable waiting times. The only hospital having CT in immediate connection to the ED was hospital A in test 2. This consumed in the other hospitals long transport times with involvement of the trauma-team staff. 

Lack of transport staff became an indirectly limiting factor in all the tests, consuming time for medically qualified staff to do transports even in cases where such competence not was required. 

### Supplies

In all these tests, lack of supplies appeared to be a problem. Most prominent was lack of disposable material for surgery, and amongst instruments, sets for external fixation. This was in the tests solved by extensive transfer of such stuff from other hospitals, and opening of commercial supplies. Whether this really would have worked to such an extent in a real situation can be discussed.

The calculated need of blood was in all tests kept within the available reserve capacity, based on efficient resuscitation and consequent use of damage control surgery. These calculations, however, had limitations: (a) They did not pay attention to the blood groups of simulated casualties, and (b) they may have been optimistic with regard to the need—single complications in trauma surgery can consume large blood volumes. And finally (c), they did not take into account the extensive need of blood after the period for the test.

### Staff evaluation of the tests

Evaluation of the methodology by the participating staff was done in immediate connection to the test in a written survey using a scale 1–10, where 1 = not at all, 10 = very much (Table 2). The accuracy of the methodology to test surge capacity was in this survey ranked as MD 8 (IQR 2). The value of the methodology to identify deficiencies and potential improvements in the disaster plans was on the same scale ranked as MD 9 (IQR 2) and the value for simultaneous training as MD 9 (IQR 3) (Table 2).

**Table 2:** Evaluation of methodology by participants

Surge capacity test	*n*	QRR	MD (IQR)	Min–max
Question 1: How relevant do you consider the used methodology for calculation of the surge capacity of your hospital?				
A1	26	77%	8 (1)	6–10
A2	97	96%	8 (1)	4–10
B1	57	95%	8 (1)	3–10
B2*	145	96%	8 (2)	5–10
B3	63	89%	8 (1)	5–10
C1*	129	100%	8 (2)	1–10
Question 2: How valuable do you consider this test to identify deficiencies or potential improvements in the hospitals preparedness?				
A1	28	82%	8 (1)	7–10
A2	101	100%	9 (2)	5–10
B1	58	97%	9 (2)	5–10
B2*	151	100%	10 (1)	6–10
B3	68	96%	9 (2)	5–10
C1*	129	100%	10 (1)	6–10
Question 3: Do you think that this exercise has improved your ability to respond accurately to a major incident in your present function?				
A1	27	79%	8 (3)	3–10
A2	101	100%	8 (3)	2–10
B1	57	95%	8 (2)	2–10
B2*	127	84%	9 (3)	5–10
B3	67	94%	9 (2)	5–10
C1*	129	100%	9 (2)	1–10

Evaluation of the methodology by the participating staff using a scale 1 – 10 where 1 = not at all, 10 = very much

*Civilian and military co-operation using live figurants transported to the hospital by the military. The figurants were treated in ED and changed to casualty cards with the same injuries for the continued processing in the hospital

### Side effects for hospital emergency plans

The disaster plans were as an effect of the test extensively revised following the oral and written comments based on the experiences from the exercise. This illustrates the need of regular exercises to test the plans.

## Discussion

With the use of this technique, it was possible to numerically determine the capacity-limiting factors for each phase of the response for all studied hospitals. The severity of the simulated mass-casualty event, overloaded all emergency departments and lead to long waiting times and the need to temporarily close the intake of more patients to the ED. None of the three hospitals´ OR capacity was shown to be exceeded for the time during which the simulation exercise was performed. All hospitals’ ICU capacity was reached after a few hours into the exercise and this was the final limiting factor. The timewise relationship between the limiting factors is coherent with previous scientific studies from real events and from previous simulation tests [22].

Thorough evaluation and dedicated time must be allocated in order for hospitals to become aware of their capacity-limiting factors and to find solutions such as using different working strategies, alternative rooms, restrictive utility use and improved systems to overcome the limiting factors.

### Need of capacity tests

A prerequisite for setting demands on hospitals and regions in the field of preparedness is to define specified goals for health care with regard to what different units should be able to handle in a sudden and unexpected high load of injured or critically ill. Such goals are set for all other areas of health care, but rarely for preparedness, where the demands usually are very fluent. This makes it difficult to (a) make necessary improvements to cope with potential needs, (b) plan distribution of patients between hospitals in an MCI, and (c) control the quality of preparedness, which became apparent during the COVID-19 pandemic.

To set and control such goals, requires methods for precise determination of capacity for different units. It is a misconception that capacity can be theoretically calculated only based on resources (facilities, equipment, staff). The capacity is also dependant on efficiency of the organisation (relevance of disaster plan) and of the competence of the staff (education and training). A well-organised unit with well-trained staff may have much higher capacity than a unit with more resources, but insufficient plan and un-trained staff. Therefore, defining of capacity requires practical tests.

### Relevance of the used technique

Test of this kind can be either done with live figurants, moulaged or with cards with description of injuries, or by different kinds of simulations. To bring in live figurants in the hospital means interfering with normal activity and is connected with very high costs. It has been shown that full-scale exercises with live figurants in a hospital have been around ten times more expensive than a corresponding simulation exercise [23]. In addition, it is very difficult to get the same amount of detailed information with live figurants as in a simulation.

However, in a new hospital, or after re-building, partly use of live figurants may be necessary in a first test, for example testing ED capacity; are the rooms planned for major trauma in an MCI suitable with regard to space and equipment? Logistics in the ED in MCI? This was how one of the tests was performed in hospital B and also in hospital C, using live figurants in ED and then changing to cards with the same injuries for the continued processing in the hospital.

The simulation system used in this study [14, 15] was initially developed for scientific analysis and comparison of different methods in MCI management [16], meaning high demands on precision. The accuracy of the system as an educational tool had been scientifically validated in an international study [17]. The fact that it was based on real casualties from real scenarios, and supplied complete information of consumption of time, staff and material for every measure performed, made it very suitable as a test instrument. Use of standardised scenarios also made the tests reproducible, making it possible to determine the effects of additional resources, revised methodology or increased training.

The reason that we adapted the total number of dead and injured to the local population was to achieve a realistic proportion between the total casualty load and the locally available resources for transportation and health care. You cannot simulate an aeroplane crash with aircraft taking maximally 100 passengers and have 500 injured and dead, like in a Jumbo jet crash. In the same way, if we—as here—simulate an attack to a communication system, we cannot have more injured and dead that possibly could be in that system on one and the same time. This would cause an unrealistic load on local transport and health care facilities, since they are adapted to the local population. This does not influence the reproducibility, since the proportions with regard to type and severity of injuries in the used scenario are maintained.

### Capacity limiting factors

These tests clearly demonstrated that there are different capacity-limiting factors in different phases of the response, and how these factors were related to each other. Knowledge of this is a prerequisite for leading and coordinating the response in an efficient way on local as well as regional level.

**Fig. 7:**
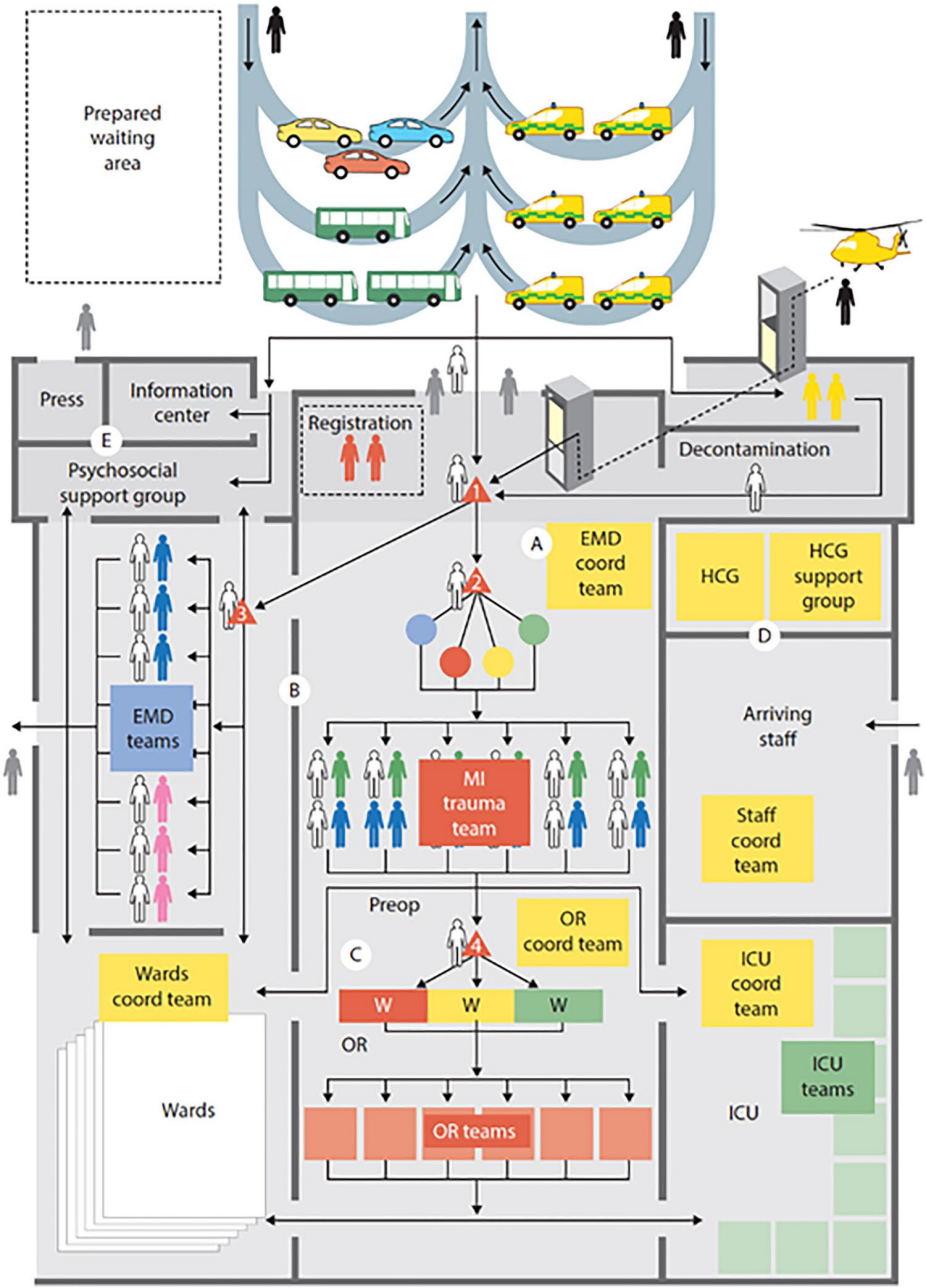
Schematic illustration of the patient flow in the hospital in MCI response. The critical point in the first phase of the response is point 2 in the figure, secondary triage of severely injured and distributing them between available “Major incident trauma-teams” (MIT, designed to achieve optimal number of parallel teams with maintained quality of care). Waiting times to these teams have to be kept at a minimum to avoid complications and mortality. A higher number of severely injured than the teams can handle simultaneously should therefore not be referred to this hospital but directed elsewhere. Less severely injures can after primary triage run in separate line, consuming less resources, and is rarely a limiting factor for hospital capacity. Modified from Lennquist S: The hospital response. In: Lennquist S (Ed): Medical response to major incidents and disasters, Springer 2012, with permission

The limiting factor for hospital capacity in the first phase of the response is the number of severely injured (major trauma) patients that the ED can handle simultaneously, without waiting times involving a risk for preventable mortality and severe complications (Fig. 4). The number of teams and facilities for this a hospital can mobilise to work parallel is limited. It requires (a) prepared rooms with sufficient space and prepared equipment for major trauma and (b) teams with training in trauma management. Even if the goal to provide complete trauma teams, as in routine trauma management, rarely can be met in MCI, it requires a minimal number of trained staff. Figure 7 illustrates schematically the patient flow in a hospital in MCI [24]. Patients with major trauma should not wait. If the waiting times at point “2” in the diagram become too long, it will result in mortality and complications, as illustrated in Fig. 4.

It is currently not fully known how many parallel “Major incident trauma teams” different Swedish hospitals can mobilise in reality. This important capacity-limiting factor demands further exploration and guidelines for this should be elaborated based on hospital size, assignment, and geographic location. The large number of teams at the first test in hospital A (Fig. 4) was exceptional and due to the fact that staff, according to the regional plan, was transferred from other hospitals. Still, it was not enough for this casualty load, leading to waiting times causing preventable mortality according to patient data provided in the system, including defined times within certain treatments had to be done to avoid mortality. Waiting times were either due to the high casualty flow, or to difficulties to restrict the time in ED, or both. One factor prolonging the time for the teams was transfer of the patients to a CT scanner outside the ED (this hospital had at that time no scanner in the ED). This tied up the teams. Access to CT in ED, and restricted use of CT, is needed to avoid this.

**Fig. 8:**
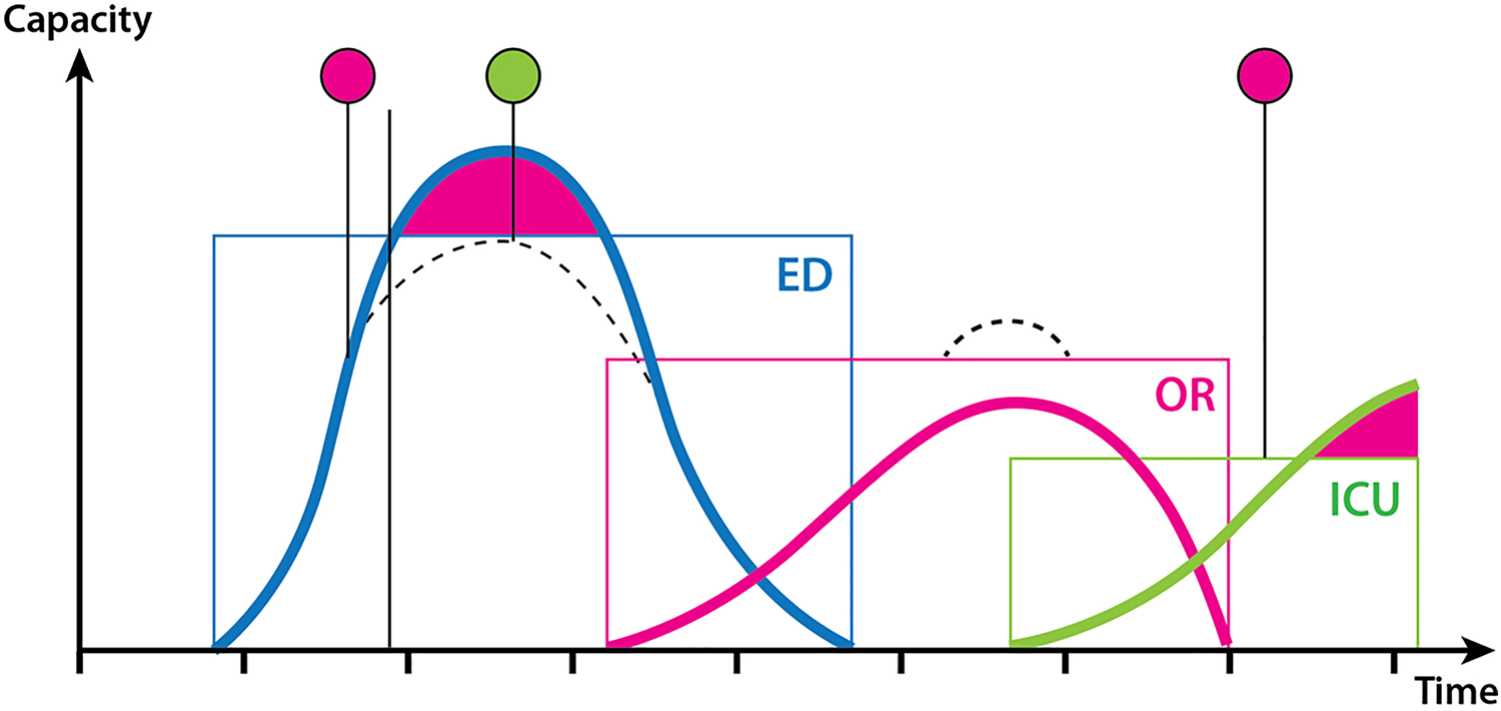
The figure illustrates the need of the coordinating functions always to be one step ahead and direct casualty flow elsewhere not when, but before the different components of the hospital are overloaded, which for different components occurs in different phases of the response (red signals). Equally important is to “open up” the flow if and when capacity is restored, which can be valid for the ED very fast and later for the OR, if overloaded. In all these tests, ICU capacity was the final limiting factor and that must be foreseen before it occurs

To avoid waiting times for severely injured potentially causing mortality, the inflow must be stopped before the capacity is exceeded. It is too late to do it when all teams for this already are fully occupied. It has to be done in advance, based on knowledge of the inflow as well as the capacity limit (Fig. 8). Equally important as stopping the inflow in these situations is to start the inflow as soon as there is again space in the ED (Fig. 8); otherwise, the hospital capacity for surgery and intensive care will not be fully utilised.

**Fig. 9:**
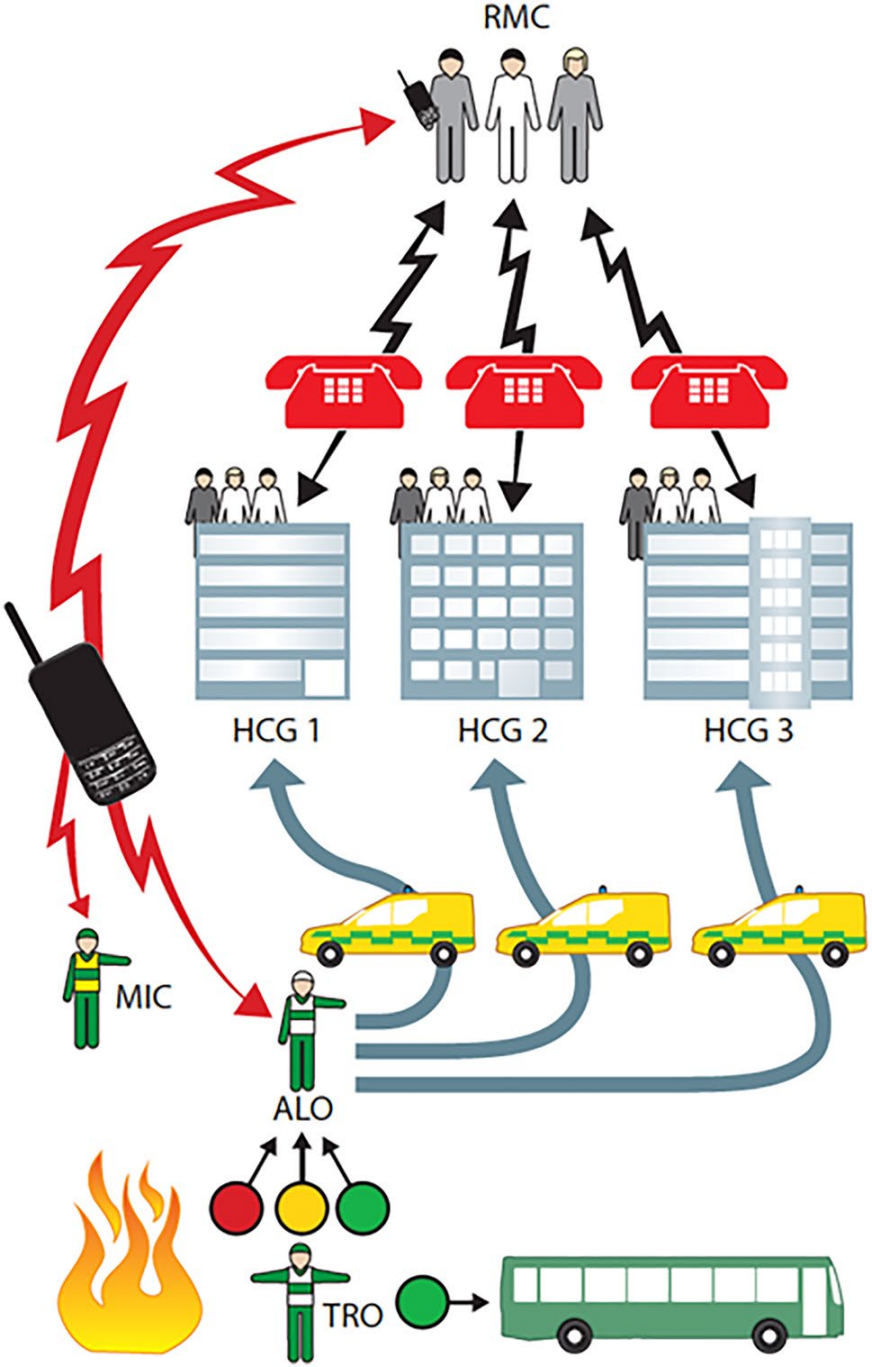
Schematic illustration of the coordination between scene (ambulance loading officer, ALO), hospitals (hospital command groups, HCG) and regional medical command centre (RMC). This coordination must be well trained and staffed by personnel with good knowledge of all components in the chain of management, staff that has to be repeatedly trained for this task and serve in an on-call system for MCI as a necessary component of the preparedness. Modified from Lennquist S, Dobson R. The prehospital response. In Lennquist S (Ed): Medical response to major incidents and disasters, Springer 2012, with permission

This requires coordination between the ambulance loading officer on scene and the Ambulance Dispatch Center in close collaboration with the regional command centre for health care, receiving continuous reports from the hospitals [25] (Fig. 9).

The surgical (OR) capacity was in none of the tested hospitals a limiting factor. All these hospitals had a very good OR capacity, but in smaller hospitals, it is most likely that the OR will become an at least temporary limiting factor in high casualty loads. Since the load on OR comes later that the load on ED, there is often time to either clear the theatres by stopping all planned surgery (during office hours, as in Fig. 6), or mobilise staff (during non-office hours). Limiting factors here were access to disposable material, and certain instruments, like sets for external fixation of fractures and (in hospitals with neurosurgery) neurosurgical sets. The last point can easily be adjusted as a measure of preparedness. The disposable material is a problem, since many hospitals today by economic reasons have restricted their supplies to “just-in-time”. The hospitals have to be requested to have supplies for MCI. Again, this requires setting clear numerical goals for preparedness.

One way to cope with the need of OR capacity in smaller hospitals is to use other rooms, for example in out-patient clinics, for minor surgery in local or regional anaesthesia. During the Tsunami disaster 2004, some of the Thai hospitals could duplicate their OR capacity in this way, partly due to the fact that many injuries were wounds suitable for this [26]. This is something that could be included in hospital preparedness.

The final capacity-limiting factor in all these tests was available ICU-beds, even if some “ordinary” ICU patients could be transferred to wards, or (if their conditions did permit) could be transferred to other hospitals. One effect of COVID-19 is that the access to ventilators “in reserve” has increased in most hospitals. However, the staff has not increased in accordance with that. In many hospitals during the pandemic, staff from OR/Anaesthesia could be used to handle extra ventilators, but in an MCI with severe trauma, this staff is needed for surgery.

The lack of ventilators must be predicted before it occurs; otherwise, it will involve a risk of losing patients, since secondary transfer of these patients is well known to involve a risk of increased mortality**.** This illustrates again the demand of coordination and always “thinking ahead”.

Access to hospital beds was in none of these tests a limiting factor. This in in agreement with the experiences from most major incidents during the last decades, even with very high casualty loads, like World Trade Center 2001[27], Madrid 2004 [21], London 2005 [2]. This may appear surprising, since lack of beds is a daily problem in most hospitals during routine medical care. However, in a full alert, much staff not on duty is called in, increasing the possibilities to receive patients, and most hospitals have a supply of extra beds. There are also patients that can be dismissed without risk of complications (planned surgery or investigations). The need to dismiss patients in these major incidents has been low, and sending home patients unnecessarily has been criticised [2]. This also emphasised that access to beds do not need to be considered in the primary capacity reports from the hospitals—the situations in ED, OR and ICU are the critical factors.

The difference in results between office- and non-office hours in these tests was mainly restricted to the ED, where mobilisation of staff naturally took more time than during office hours. This requires special attention in the first distribution of severely injured between hospitals. For OR and ICU, no significant differences were registered. This can be explained by the fact that this need comes later in the response. According to the alert tests done before the non-office hour test, the majority of staff not on duty were available and could be in the hospital within half to 1 h after the alert. This was enough to staff a sufficient number or theatres and extra ventilators within the time-limit required. This is also in accordance with experiences from major incidents where most of the staff often come in, also spontaneously, very fast after an MCI has occurred, and the problem has sometimes been over-staffing. Special occasions, like holidays, may of course still cause staff problems.

Participating staff of all categories ranked this methodology very suitable for test of hospital capacity. They also ranked the value of the test for training as very high. Since the tests can be done with very limited costs, and without interfering with routine medical care, they can be recommended as an efficient combined tool for training and quality assurance.

## Limitations

Simulation exercises carry inherent and obvious limitations with regard to the transferral of the results from a simulation upon the evaluation of real resources, structures and systems. Part of this can be overcome using detailed and advanced simulations such as described in this study.

Further limitation of this study was that it was restricted to hospitals with relatively good access to surgical theatres and intensive care beds. In medium size or smaller hospitals, surgical capacity will probably be a capacity-limiting factor in a second phase of the response, unless lack of ICU facilities stops surgery even before its limit is reached. This requires studies extended also in minor hospitals. Another limitation is that the capacity was tested for only one kind of scenario, incidents caused by physical violence. The simulation system used includes also other scenarios (burns, hazmat) which opens for further studies.

## Conclusions

These tests illustrated:

- How the capacity-limiting factors for hospitals in an MCI vary for different phases of the response, and how these factors interfered with each other
- The need of continuous and immediate coordination of the distribution of casualties between hospitals, and the need for the coordinating units always to be “one step ahead”
- Possible measures to increase the capacity on different points in the chain of response
- How capacity tests can be done for reasonable costs and be combined with efficient training of staff of all categories.
