# Correction: A method for detailed determination of hospital surge capacity: a prerequisite for optimal preparedness for mass-casualty incidents

**DOI:** 10.1007/s00068-022-02133-4

**Published:** 2023-01-04

**Authors:** Kristina Lennquist Montán, Per Örtenwall, Magnus Blimark, Carl Montán, Sten Lennquist

**Affiliations:** 1 Department of Global Public Health, Karolinska Institute, Solna, Sweden; 2 University of Gothenburg, Göteborg, Sweden; 3 Centre for Defence Medicine, Swedish Armed Forces, Göteborg, Sweden; 4 Department of Vascular Surgery, Karolinska Institutet, Stockholm, Sweden; 5 University of Linköping, Linköping, Sweden

**Correction: European Journal of Trauma and Emergency Surgery** 10.1007/s00068-022-02081-z

In this article the presentation of Figs. 1 and 4 were incorrect and the legend of Fig. 4 was incomplete. The Fig. 1 and 4 should have appeared as shown below.

**Fig. 1:**
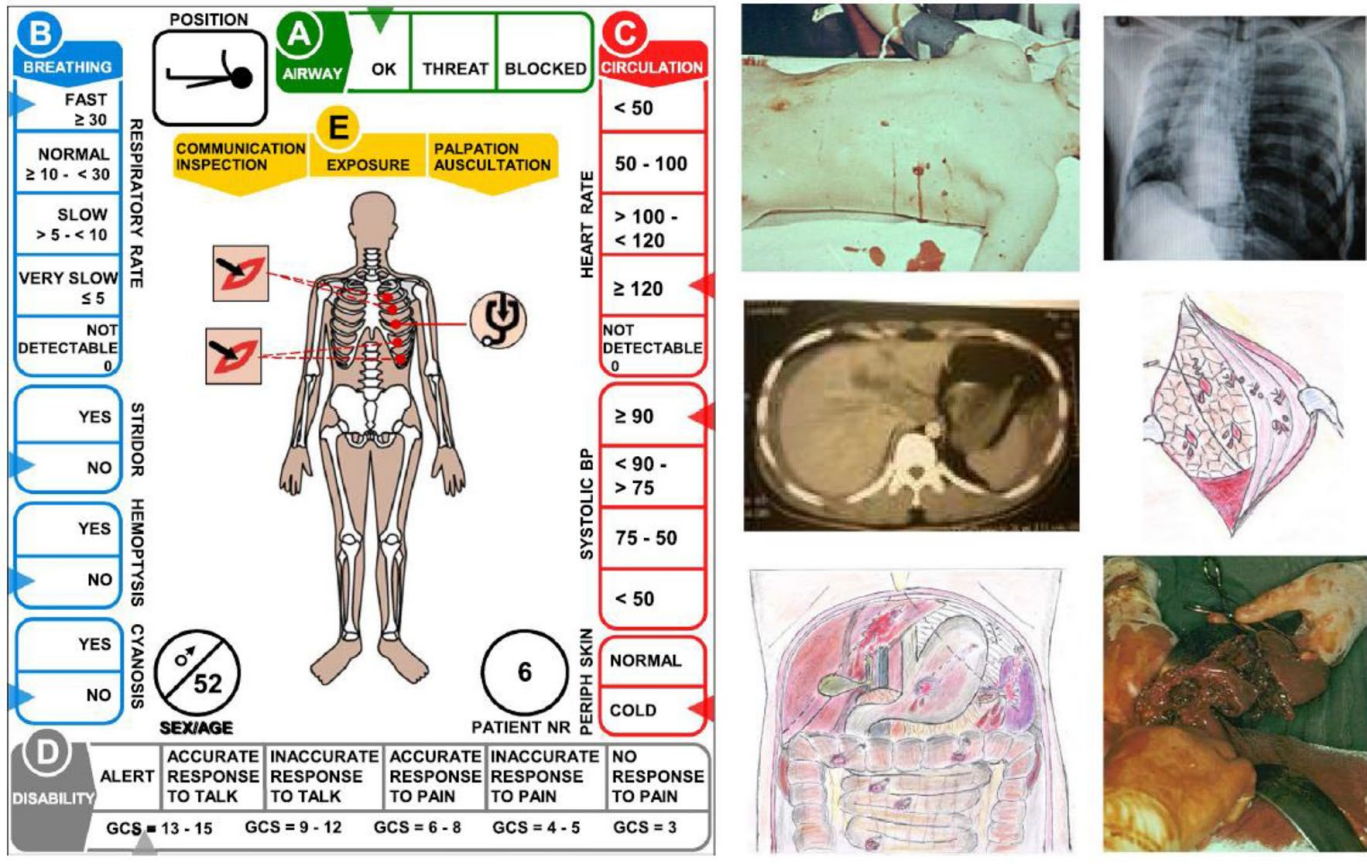
The casualty cards used in this study (for description, see text) were based on real patients from real scenarios. The cards were connected to data files with live pictures, X-ray—and surgical findings as base for decisions with regard to treatment. For each patient, the instructors had access data regarding treatments that had to be done within a certain time to avoid mortality. This made it possible to determine the outcome as a result of the response and of the methodology used

**Fig. 4:**
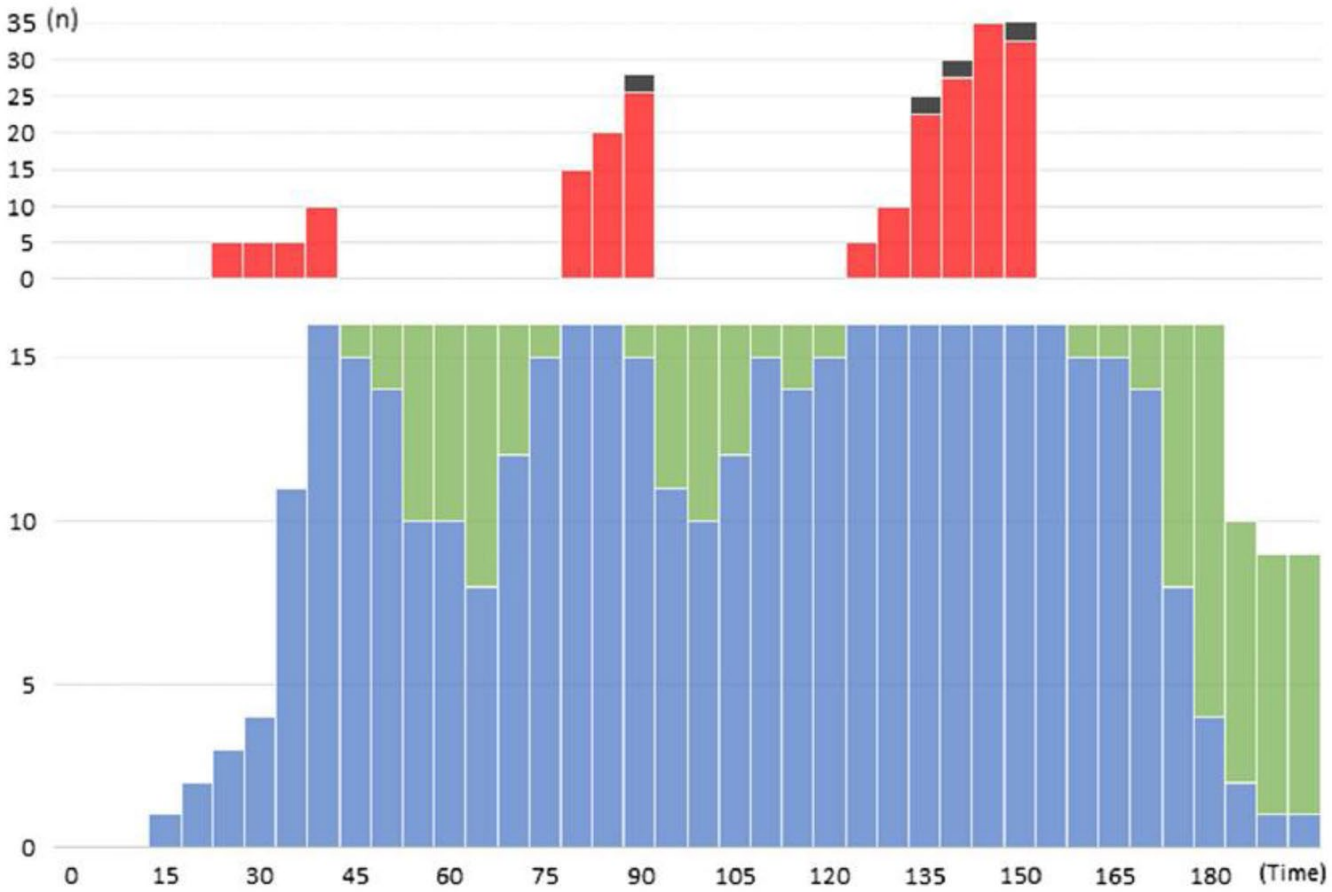
The activation and use of the teams for management of severely injured casualties in the Emergency Department (ED) in one of the tested hospitals (see further the text). The periods of very high casualty load, causing waiting times leading to calculated mortality, correspond to the “waves” of ambulances between returning and re-loading. To avoid preventable mortality, the inflow has to be temporarily stopped and casualties referred elsewhere. This puts high demands on coordination of casualty distribution. Blue: Trauma-teams (modified for MCI) in action, Green: Such trauma teams at disposal, Red: Severely injured patients having to wait for access to teams, Black: Preventable deaths caused by waiting

The original article has been corrected.

